# The Pharmacokinetics of Intravenous and Subcutaneous Ondansetron in Female Beagle Dogs

**DOI:** 10.1111/jvp.70058

**Published:** 2026-02-16

**Authors:** Elena D. Landau, Kendal R. Boege, Kristen Messenger, Gregory Ballash, Daniel Gustafson, Carolina Ricco Pereira, Krista Banks, Turi Aarnes, Phillip Lerche, Monica Midon, Kirk A. Muñoz

**Affiliations:** ^1^ College of Veterinary Medicine The Ohio State University Columbus Ohio USA; ^2^ Biomedical Sciences and Pathology Virginia‐Maryland College of Veterinary Medicine, Virginia Tech Blacksburg Virginia USA; ^3^ Veterinary Preventive Medicine, College of Veterinary Medicine The Ohio State University Columbus Ohio USA; ^4^ Clinical Sciences Department Colorado State University Fort Collins Colorado USA; ^5^ Veterinary Clinical Sciences, College of Veterinary Medicine The Ohio State University Columbus Ohio USA

**Keywords:** canine, intravenous, ondansetron, pharmacokinetics, subcutaneous

## Abstract

The purpose of this study was to evaluate the pharmacokinetic profile of ondansetron following intravenous (IV) and subcutaneous (SC) administration in five healthy adult female Beagles. Dogs were administered ondansetron at 0.5 mg/kg IV and SC in a randomized crossover design. On day 0 ondansetron was administered either IV (OV group) or SC (OS group), and 7 days later administered via the opposite route. Plasma samples were collected, and heart and respiratory rates, and rectal temperature were recorded over an 8 h period. Ondansetron concentrations were quantified using liquid chromatography–tandem mass spectrometry. Non‐compartmental analysis was performed using commercially available software. Median (min—max) peak plasma concentration following OS was 84.6 (56.0–326.1) ng/mL, which occurred at the first sampled time point of 0.25 (0.25–0.5) h. The terminal half‐life was longer in the OS versus the OV group. Bioavailability of ondansetron in the OS group was 84.6 (51.2–132.6)%. Ondansetron administered SC at 0.5 mg/kg to adult healthy dogs has a similar bioavailability and a longer duration of action compared to the IV route of administration. Future pharmacokinetic and pharmacodynamic clinical studies should be performed in dogs utilizing the SC route of administration of ondansetron.

## 1 Introduction

Medications administered during the perianesthetic period, such as opioids, can increase the risk of nausea and vomiting (Ryan et al. 2022). These risks can result in aspiration, discomfort, and if severe, death (Foth et al. 2021; Moyer et al. 2021). Maropitant is frequently used to reduce the incidence of vomiting in dogs, but injectable maropitant's expense and limited anti‐nausea efficacy at the recommended dosage of 1 to 2 mg/kg reduces its utility in dogs (Burke et al. 2021; Kenward et al. 2017).

Ondansetron, a serotonin (5HT3) receptor antagonist, offers a cost‐effective alternative due to its combination of both anti‐nausea and antiemetic properties. In cats, subcutaneous administration at a dose of 2 mg/cat (mean dose 0.4 mg/kg) has been shown to produce prolonged therapeutic serum concentrations (estimated to be between 25 and 75 ng/mL in cats) compared to intravenous and oral routes, suggesting a favorable pharmacokinetic profile for clinical use when IV access is not readily available (Quimby et al. 2014). In contrast, oral administration in dogs has demonstrated limited efficacy and absorption. Plasma concentrations of oral ondansetron were below detectable limits in hospitalized canine patients, indicating poor bioavailability and raising concerns about its clinical utility (Zersen et al. 2024). These limitations were further highlighted in a randomized study in healthy dogs, where oral ondansetron administered prior to premedication was less effective at reducing vomiting compared to oral maropitant (Burke et al. 2021).

Given the limited efficacy and poor bioavailability of orally administered ondansetron in dogs, alternative routes of administration have been explored. A study examining the pharmacokinetics of intravenously administered ondansetron in sick dogs found that IV administration of 0.5 mg/kg ondansetron every 8 h resulted in therapeutic plasma concentrations (estimated to range between 10 and 250 ng/mL) and a reduction in nausea scores in the majority (83%) of patients, highlighting ondansetron's use as an effective antinausea medication (Sotelo et al. 2022). However, to date, there are no published data on the pharmacokinetics and bioavailability of subcutaneously administered ondansetron in dogs.

The objective of this study was to determine the plasma concentrations and pharmacokinetic profiles of ondansetron following IV or SC administration at a dosage of 0.5 mg/kg in healthy adult dogs. This dosage was selected based on previous studies in cats and dogs, as well as commonly used clinical doses in dogs, reflecting a practical and effective therapeutic regimen (Quimby et al. 2014; Sotelo et al. 2022).

## 2 Materials and Methods

### 2.1 Animals

Five healthy purpose‐bred Beagle dogs were used in this study. The cohort included four spayed females and one intact female, all aged 5 years, with body weights ranging from 8 to 10 kg. The study was approved by the Institutional Animal Care and Use Committee (IACUC) #2023A00000069 at The Ohio State University. Dogs were housed together at The Ohio State University's research facility, where they received daily care and enrichment from their caretakers. On each study day, two dogs were transported to the laboratory where drug administration and sampling were performed and then returned to their housing at the end of the day. Dogs were provided with water all day but not fed until they returned to the research facility at the end of the day. All dogs were adopted at the conclusion of the study.

### 2.2 Drug Administration

This was a prospective, randomized, crossover study. Dogs were administered ondansetron (2 mg/mL) (Fosun Pharma USA Inc., Princeton, NJ) intravenously (OV group) and subcutaneously (OS group), both at a dosage of 0.5 mg/kg, on separate occasions with a seven‐day washout period between groups.

### 2.3 Dosing and Sampling

Two 20‐gauge 1 IV catheters (Terumo Medical Products, Maryland) were aseptically placed in both cephalic veins for dogs in the OV group; one for ondansetron administration and the other for blood sampling, while dogs in the OS group only had a single IV catheter placed for blood sampling. The IV catheters were flushed with saline prior to and after blood sampling at each time point. To prevent dilution from residual saline flush, a waste volume of blood was drawn from the IV catheter prior to sample collection. A bite guard was placed over the IV catheters to prevent the dogs from damaging them. Ondansetron was administered on the dorsal aspect of the back between the shoulder blades using a 22 G 1.0″ hypodermic needle (Medline Industries, Northfield, IL) in dogs in the OS group.

Vital parameters, which included rectal temperature and pulse and respiratory rates, were recorded as a baseline prior to ondansetron administration, and then at 1, 2, 4, 6 and 8 h. Pulse rate was determined by palpation of a femoral pulse and counted over a period of 30 s. Respiratory rate was determined by observing chest excursions and counting over a period of 30 s. Blood samples were collected at baseline (−0.08), 0.25, 0.5, 1, 2, 4, 6 and 8 h after ondansetron administration. Blood was placed in EDTA blood collection tubes and stored on ice until they were separated by centrifugation at 1000 *g* for 10 min at 21°C (Centrifuge 5810 R 15‐amp version, Eppendorf AG). Plasma was harvested and stored at −80°C until batch analysis. The SC site of ondansetron administration was evaluated hourly for the first 8 h, and then 24 h later for signs of redness and swelling. Gentle palpation around the site of administration was also performed every hour during the first 8 h to check for signs of pain.

### 2.4 Analytical Methods

Plasma concentrations of ondansetron were quantified using liquid extraction followed by liquid chromatography–tandem mass spectrometry (LC–MS/MS) analysis. Stock solutions of ondansetron (Sigma Aldrich, 1478571) and zolpidem (Sigma Aldrich, Z‐103) were prepared at 1 mg/mL in methanol and stored at −20°C. Calibration standards were prepared by spiking blank canine plasma (100 μL) with 10 μL of 10‐fold concentration ondansetron reference standard solution and 10 μL of 10‐fold concentration internal standard (zolpidem, 100 ng/mL) in 2.0 mL polypropylene microtubes. All other chemicals were purchased from Sigma Aldrich. A 1.0 mL volume of 50:50 ethyl acetate: pentane containing 0.1% ammonium hydroxide was added to each tube. Final ondansetron concentrations in the standard curve ranged from 0.98 to 2000 ng/mL (0.98, 1.95, 3.9, 7.8, 15.6, 31.25, 62.5, 125, 250, 500, 1000, and 2000 ng/mL). Quality control (QC) samples were prepared in quadruplicate at final concentrations of 5, 50, and 500 ng/mL using the same procedure. All tubes were vortexed on a shaker at room temperature for ≥ 10 min, then centrifuged for 10 min at room temperature. Following centrifugation, 800 μL of the organic phase was transferred to clean, labeled polypropylene tubes and evaporated to dryness on a RapidVap concentrator under gentle nitrogen flow at 60°C for approximately 30 min. Dried residues were reconstituted in 100 μL of 50:50 methanol: 5 mM ammonium acetate with 0.1% acetic acid and shaken at room temperature for 1 h. After centrifugation, reconstituted samples were transferred to LC–MS autosampler vials with polypropylene inserts.

Canine plasma samples were extracted using the same protocol. Each 2.0 mL polypropylene tube received 100 μL of plasma, 10 μL of internal standard, and 10 μL of 50:50 methanol:MilliQ prior to the addition of the organic extraction solvent (Sotelo et al. 2022; Garrick et al. 2025). All samples were run as a single batch so there was no need to take inter‐day reproducibility into consideration.

### 2.5 Ultra‐Performance Liquid Chromatography–Tandem Mass Spectrometry (UPLC/MS/MS) Analysis

Ondansetron plasma concentrations were quantified using a Thermo TSQ Quantis Triple Quadrupole Mass Spectrometer coupled to a Vanquish UHPLC pump system with an integrated autosampler. Chromatographic separation was performed using a Phenomenex Kinetex Phenyl Hexyl column (2.5 μm, 4.6 × 50 mm) with a Phenomenex C18 Filter Frit Guard Cartridge. The column oven was maintained at 30°C. The mobile phases consisted of methanol (organic phase) and 10 mM ammonium acetate with 0.1% acetic acid (aqueous phase). The LC gradient began with 10% methanol and 90% aqueous buffer, ramped to 98% methanol by 2.0 min, held until 3.5 min, and then returned to initial conditions by 4.0 min. The total running time was 5 min. The flow rate was 900 μL/min and the injection volume was 1 μL.

Mass spectrometry was performed in selected reaction monitoring (SRM) mode using positive ionization. Instrument parameters specific to each compound were defined in the method addendum (Appendix S1). Ondansetron and the internal standard zolpidem were monitored throughout.

Working solutions for calibration curves and QC samples were prepared in 50:50 methanol: water. The r2 value for calibration curves was > 0.99 and batch acceptance was based on > 75% of QC samples having an accuracy of ≥ 85%. The lower limit of quantification (LLOQ) for the analysis was 0.98 ng/mL based on the lowest concentration at least twofold above baseline and having an accuracy > 80%.

### 2.6 Statistical Analysis and Pharmacokinetic Methods

Pharmacokinetic parameters were calculated using a non‐compartmental analysis with Phoenix WinNonlin v8.5.2.4 (Certara, Princeton, NJ). The linear up log down method was used to calculate the area under the concentration‐time curve (AUC), and values for the maximum plasma concentration (*C*_max_) and time to maximum plasma concentration (*T*_max_) were taken directly from the data. Other parameters estimated included terminal half‐life (*t*_1/2_ λ), mean residence time (MRT), volume of distribution (*V*_*z*_ and *V*_ss_), and clearance (CL). Bioavailability (*F*%) was calculated as (AUC_SC/AUC_IV) × (Dose_IV/Dose_SC) × 100. An analysis of variance (ANOVA) was used to analyze pulse (PR) and respiratory (RR) rates and rectal temperature (RT), and significance was considered at *p* < 0.05. Data for PR, RR, and RT were normally distributed and presented as mean ± SD, while pharmacokinetic data were not normally distributed and presented as median (interquartile range). The Shapiro–Wilk test was used to determine normality of the data. Descriptive statistics were used to report both OV and OS group data. Relevant pharmacokinetic parameters were compared between routes using a Wilcoxon Signed Rank test, and significance was considered *p* < 0.05.

A sample size of five dogs was selected based on previous studies in which similar numbers of dogs and cats were used in pharmacokinetic studies (Jay et al. 2013; Quimby et al. 2014; Wetzel et al. 2024).

## 3 Results

### 3.1 Drug Administration

There were no signs of discomfort, crying, or adverse effects in 4 of the 5 dogs at the time of SC injection or during the post‐administration monitoring period. One dog in the OS group vocalized and jumped during the injection, but the reaction resolved quickly with no further complications. No dogs exhibited any adverse reactions at the SC injection site during the 8 h data collection period. The SC injection site was evaluated 24 h after administration in all five dogs, with no evidence of local irritation noted. Anecdotally, dogs that exhibited signs of nausea, such as lip licking and drooling, during transport to the study site showed noticeable clinical improvement within 5 min of IV administration and within 15 min following SC administration.

### 3.2 Ondansetron Pharmacokinetics

**FIGURE 1:**
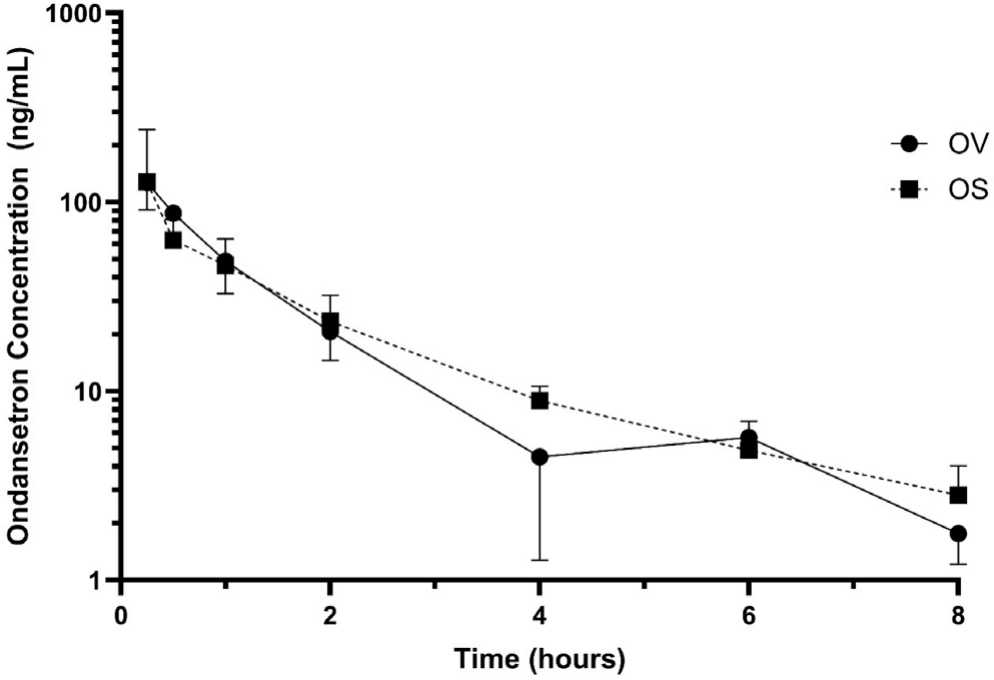
Mean (±SD) plasma ondansetron concentrations (ng/mL) in dogs administered a single 0.5 mg/kg dose of ondansetron intravenously (OV group) and subcutaneously (OS group).

**FIGURE 2:**
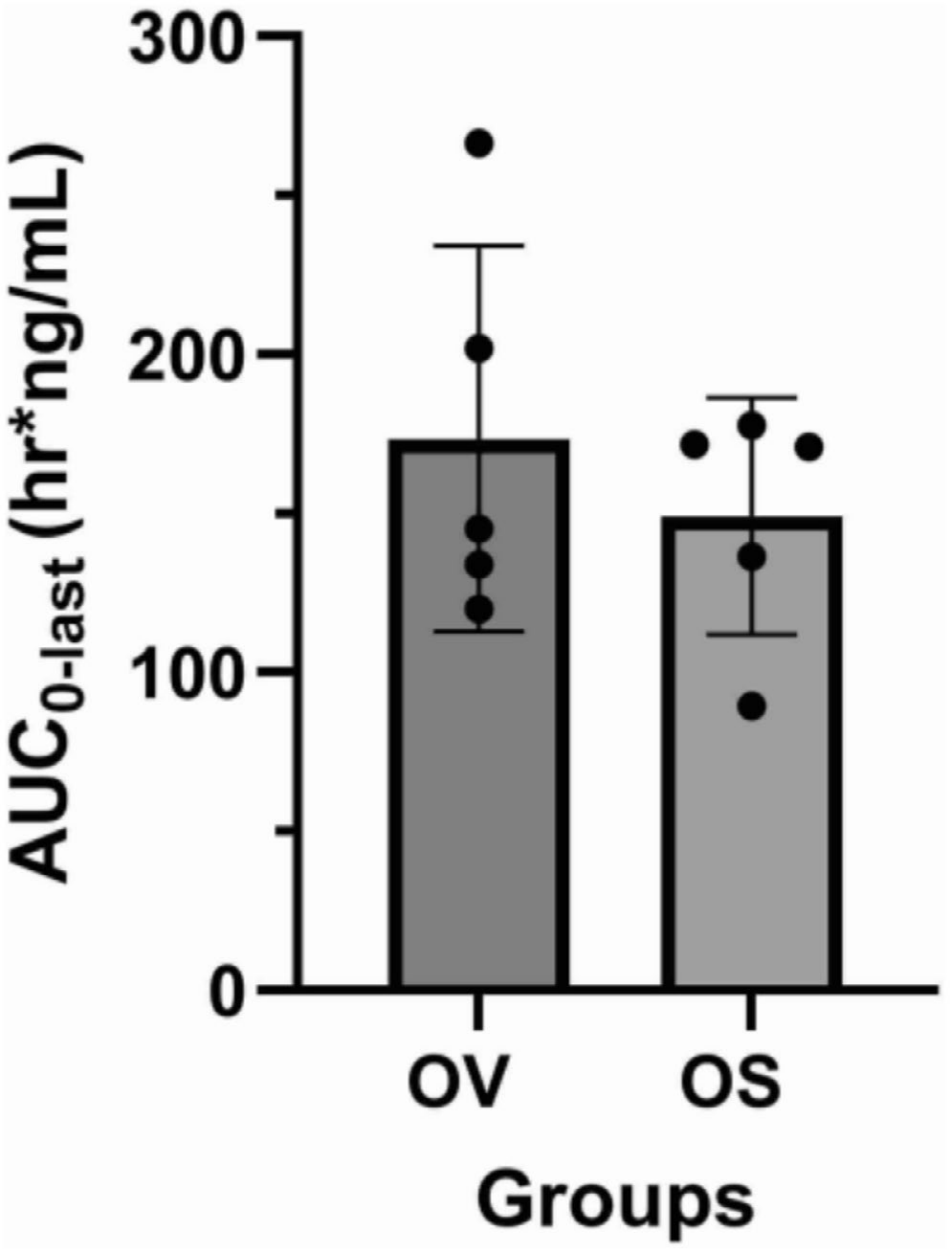
AUC_0‐last_ values (ng/mL × min) for the intravenous (OV) and subcutaneous (OS) groups. There was no significant difference between the intravenous and subcutaneous routes of administration of ondansetron at 0.5 mg/kg to healthy dogs (*p* = 0.63).

Ondansetron pharmacokinetics were similar between the OV and OS groups (Figure 1 and Table 1). Compared to the OV group, the OS group had a slightly longer *t*_1/2_ λ (1.3 vs. 1.9 h, respectively), although these differences were not significant (*p* = 0.06). Plasma concentrations were not significantly different between groups at the first sampled time point of 15 min (median 127 ng/mL for OV and 85 ng/mL for OS; *p* = 0.62), which was the median *T*_max_ for the OS group (min–max 15–30 min). The area under the curve (AUC_0‐last_) (Figure 2) was also not different between groups (*p* = 0.63). Clearance following IV administration was 57 (31–69) mL/min/kg, and the volume of distribution at steady state (*V*_ss_) was 4.3 (3.5–6.7) L/kg. Bioavailability (*F*%) in the OS group was found to be 84.6 (51.2–132.6). The accuracy and the precision (RSD%) were 93.2% ± 2.8%.

**TABLE 1:** Pharmacokinetic parameters following 0.5 mg/kg ondansetron intravenously (OV) and subcutaneously (OS) in healthy Beagle dogs (*n* = 5). Data are presented as median (min—max).

Parameter	OV group	OS group
*C*_max_ (ng/mL)	N/A	84.6 (56.0–326.1)
*T*_max_ (h)	N/A	0.25 (0.25–0.5)
*t*_1/2_ *λ* (h)	1.3 (1.1–1.8)	1.9 (1.2–2.7)
AUC_0‐last_ (h × ng/mL)	145.1 (119.9–266.4)	170.9 (89.4–177.7)
AUC_0‐inf_ (h × ng/mL)	146.4 (120.6–269.3)	179.7 (94.4–184.6)
AUC % extrapolated (%)^a^	1.1 (0.7–2.4)	6.5 (1.1–7.6)
AUMC_last_ (h × h × ng/mL)	186.1 (138.1–478.7)	302.3 (179.5–346.8)
MRT_last_ (h)	1.6 (1.0–1.8)	2.0 (1.6–2.2)
*V*_*z*_ (L/kg)	6.1 (3.6–8.7)	N/A
*V*_ss_ (L/kg)	4.3 (3.5–6.7)	N/A
Cl (mL/min/kg)	56.9 (31.0–69.1)	N/A
Bioavailability (%)	N/A	84.6 (51.2–132.6)

Abbreviations: AUC, area under the curve; AUMC, area under the moment curve; Cl, total body clearance; *C*_max_, maximum concentration; MRT, mean residence time; *t*_1/2_ λ, terminal half‐life; *T*_max_, time to maximum concentration; *V*_ss_, volume of distribution at steady state; *V*_*z*_, the apparent volume of distribution during the terminal elimination phase.

^a^ AUC extrapolated = AUC_*t*‐∞_/AUC_0‐∞_.

### 3.3 Physiologic Variables

There were no differences between the OV and OS groups with respect to pulse rate, respiratory rate, and rectal temperature between time points over the 480 min of data collection (*p* = 0.13, *p* = 0.20, *p* = 0.54, respectively) (Table 2). There were no indications of irritation, redness, pain, or swelling at the site of SC drug administration over a 24‐h period.

**TABLE 2:** Mean (±SD) of pulse and respiratory rates, and rectal temperature of female Beagle dogs that received 0.5 mg/kg of ondansetron intravenously (OV) and subcutaneously (OS).

Time (hours)	Pulse rate (bpm)		Respiratory rate (brpm)		Rectal temperature (°F)	
	OV	OS	OV	OS	OV	OS
0	103 ± 20	96 ± 8	29 ± 5	28 ± 5	101.7 ± 0.4	101.6 ± 0.4
1	86 ± 5	98 ± 4	24 ± 3	30 ± 6	101.5 ± 1.5	101.3 ± 0.3
2	90 ± 7	84 ± 5	26 ± 2	23 ± 6	100.5 ± 0.4	100.6 ± 0.4
4	94 ± 10	87 ± 16	25 ± 5	23 ± 3	101.0 ± 0.3	100.9 ± 0.5
6	86 ± 7	87 ± 8	26 ± 6	22 ± 6	100.4 ± 0.9	101.1 ± 0.5
8	80 ± 8	89 ± 6	26 ± 2	26 ± 3	100.2 ± 0.8	100.8 ± 0.6

*Note:* *p* > 0.05 in all groups.

## 4 Discussion

Subcutaneous administration of ondansetron in healthy female dogs resulted in high plasma concentrations, with a bioavailability of 84.6%, indicating very good absorption and suggesting that SC delivery is a reasonable alternative to IV administration. This is a clinically relevant finding, as SC administration may offer a practical alternative in settings where IV access is limited or unnecessary. Although the absorption phase could not be estimated in this study due to sampling time points utilized, it appears that SC ondansetron is rapidly absorbed and has a relatively short *t*_1/2_ *λ* of almost 2 h in healthy female Beagle dogs. These data support the use of SC administration in dogs, which is clinically important since oral ondansetron in dogs has poor absorption (bioavailability ~5%) and plasma concentrations are frequently undetectable (Garrick et al. 2025).

The higher apparent SC bioavailability observed in these healthy female Beagle dogs compared with that previously reported in healthy cats likely reflects interspecies differences in subcutaneous tissue perfusion, hepatic drug clearance, and variability in SC absorption. Dogs generally demonstrate more consistent SC absorption and lower interindividual variability, whereas cats are known to exhibit greater variability in SC drug disposition (Court 2013; Quimby et al. 2014).

Plasma concentrations at the first sampled time point (0.25 h) were similar between groups despite the different routes of administration. The slightly prolonged absorption following SC administration may account for the slightly longer *t*_1/2_ *λ* observed in this group and may support longer lasting antiemetic effects for outpatient management in which IV access is limited, however, determining the effective plasma concentrations or duration of action for antinausea effects was outside the scope of this study. It is also important to note that our first sample from the IV group was collected at 0.25 h, when the drug had likely undergone some degree of redistribution or even elimination, which might have affected some of the estimated parameters. For example, the predicted initial concentrations extrapolated to time zero in the IV group were approximately double those reported at the 0.25 h time point in our study but they could have been higher if sampling had occurred earlier. Sotelo et al. (2022) also sampled at similar time points as in the present study, and reported approximately double the plasma concentration at 15 min but those animals were hospitalized for a variety of illnesses, and therefore differences in drug distribution or clearance in sick dogs could account for these differences in plasma concentrations (Sotelo et al. 2022).

Indeed, following IV administration, we report a rapid clearance of approximately 57 mL/min/kg, which is similar to hepatic blood flow. While the recent study by Garrick et al. did not directly report clearance or volume of distribution, we calculated an approximate clearance of 14 mL/min/kg following 1 mg/kg ondansetron IV in client‐owned healthy dogs (Garrick et al. 2025). The study by Sotelo and others reported an estimated clearance of 33–34 mL/min/kg following both 0.5 mg/kg and 1.0 mg/kg IV in hospitalized dogs, suggesting linear kinetics (Sotelo et al. 2022). The differences in values between studies are interesting and could be caused by breed and health differences (purpose‐bred female Beagle dogs versus client‐owned dogs of different breed and health status), but require further investigation. When volume of distribution (*V*_*z*_) was compared between studies, we report a slightly higher value (6.1 L/kg, median) to that estimated by Sotelo (average 4.4 L/kg (min–max 1.1–17.8) for both 0.5 and 1 mg/kg IV), but overall our estimates fell within a narrower range (Sotelo et al. 2022).

Dose interval modeling based on observed pharmacokinetic parameters suggests that a single SC dosage of 0.5 mg/kg ondansetron maintains plasma concentrations above the lower limit of quantification for approximately 4 to 6 h. This is consistent with findings in cats, where twice daily SC dosing could not maintain continuous therapeutic concentrations throughout the dosing period (Quimby et al. 2014). In dogs, the relatively short *t*_1/2_ λ observed following SC administration further support the need for more frequent dosing. These findings are clinically relevant in settings such as perioperative care or in treatment and prevention of opioid‐induced nausea (Ryan et al. 2022; Sotelo et al. 2022).

The results of this study support subcutaneous administration of ondansetron as an alternative route for administration in dogs. Its high bioavailability makes ondansetron useful in settings where IV access is limited, such as in outpatient care. This route will likely provide a reliable alternative for managing nausea, especially because oral absorption is poor and unpredictable, but further pharmacodynamic studies should be performed using this regimen to determine its effect on treating signs of nausea (Garrick et al. 2025). Subcutaneous ondansetron could be clinically beneficial for managing cases such as postoperative or chemotherapy‐induced nausea and/or vomiting in dogs, especially in cases where IV access is limited and anti‐nausea or antiemetic intervention is essential (Ryan et al. 2022; Kenward et al. 2017; Zersen et al. 2024).

This study was conducted in healthy adult female Beagle dogs under controlled conditions, which may not reflect the pharmacokinetic behavior of ondansetron in clinically ill or dehydrated patients, as demonstrated in the small clinical trial by Sotelo et al. (2022). Dogs of different breeds or those with comorbidities such as hepatic or renal dysfunction may exhibit altered pharmacokinetics. The small sample size may limit how broadly these findings can be applied and may have contributed to variability in parameters such as *C*_max_ and AUC following SC administration. This study only looked at single dosing; repeated dosing and pharmacodynamics were not assessed.

This study is the first to characterize the pharmacokinetics and bioavailability of subcutaneous ondansetron in healthy dogs. A single 0.5 mg/kg SC dosage resulted in rapid absorption, high plasma concentration, and a bioavailability of 84.6%, closely mirroring that of intravenous administration. The extended *t*_1/2_ *λ*, ease of administration, and low cost make SC ondansetron a valuable treatment option, especially in cases where IV access is limited. These findings support the use of SC ondansetron as a practical and effective route for managing nausea in veterinary patients.

## Author Contributions

E.D.L.: study design, data collection, preparation of manuscript. K.R.B.: study design, data collection, preparation of manuscript. K.M.: data analysis, interpretation, visualization, manuscript preparation and review. G.B.: data analysis. D.G. data analysis, interpretation. C.R.P: study design, manuscript preparation and review. K.B.: data processing, data analysis, manuscript review. T.A., P.L., M.M: manuscript preparation and review. K.A.M: study design, data collection, interpretation, resources, manuscript preparation and review. All authors have read and approved the final manuscript.

## Funding

The authors have nothing to report.

## Ethics Statement

The authors declare that this study was approved by and conducted in accordance with the requirements of The Ohio State University Institutional Animal Care and Use Committee (#2023A00000069). The authors confirm that they have adhered to US standards for the protection of animals used for scientific purposes.

## Conflicts of Interest

The authors declare no conflicts of interest.

## Supporting information


**Appendix S1:** Supporting Information.

## Data Availability

The data that support the findings of this study are available from the corresponding author upon reasonable request.
