# Supplementary material for: The Pharmacokinetics of Intravenous and Subcutaneous Ondansetron in Female Beagle Dogs
Source: J Vet Pharmacol Ther. 2026 Feb 16;49(4):361–6. doi: 10.1111/jvp.70058 (PMC13371833; doi:10.1111/jvp.70058)
Supplement: Supplementary file 1 — Appendix S1: Supporting Information. [file JVP-49-361-s001.docx]

Thermo Scientific SII for Xcalibur Method

---- Overview ----

Name: New Instrument Method Comment:

Run time: 5.000 [min]

Instrument: VANQUISH on thermo-ttm22adc Description:

Script

initial Instrument Setup

ColumnComp.CC.Mode: StillAir ColumnComp.CC.ReadyTempDelta: 0.50 [°C] ColumnComp.CC.TempCtrl: On ColumnComp.CC.Temperature.Nominal: 30.00 [°C]

ColumnComp.CC.EquilibrationTime: 1.0 [min]

SamplerModule.Sampler.DrawSpeed: 0.200 [µl/s]

SamplerModule.Sampler.DispenseSpeed: 1.000 [µl/s]

SamplerModule.Sampler.PunctureOffset: 100 [µm]

SamplerModule.Sampler.WashTime: 4.0 [s]

SamplerModule.Sampler.WashSpeed: 50.0 [µl/s] SamplerModule.Sampler.InjectWashMode: Both SamplerModule.NeedleHeight: Safe SamplerModule.Sampler.Pump: "Pump" SamplerModule.Sampler.InjectMode: Normal SamplerModule.TempCtrl: On SamplerModule.Temperature.Nominal: 23.0 [°C] PumpModule.Pump.%B_Selector: %B3 PumpModule.Pump.%A_Selector: %A2 PumpModule.Pump.%A1_Equate: "Water + 0.1% Formic Acid"

PumpModule.Pump.%A2_Equate: "10mM Ammonium Acetate + 0.1% Acetic Acid" PumpModule.Pump.%A3_Equate: "Water + 0.1% Formic Acid" PumpModule.Pump.%B1_Equate: "Acetonitrile" PumpModule.Pump.%B2_Equate: "Acetonitrile + 0.1% Formic Acid" PumpModule.Pump.%B3_Equate: "Methanol" PumpModule.Pump.Pressure.LowerLimit: 0 [psi]

PumpModule.Pump.Pressure.UpperLimit: 14504 [psi]

PumpModule.Pump.MaximumFlowRampUp: 1.00 [ml/min²]

PumpModule.Pump.MaximumFlowRampDown: 3.00 [ml/min²]

| 0.000 | [min] | Inject Preparation  Wait ColumnComp.Ready And SamplerModule.Sampler.Ready And PumpModule.Pump.Ready |
| --- | --- | --- |
| 0.000 | [min] | Inject |
|  |  | SamplerModule.Sampler.Inject |
| 0.000 | [min] | Start Run |
|  |  | ColumnComp.CC_Temp.AcqOn |
|  |  | PumpModule.Pump.Pump_Pressure.AcqOn |
| 0.000 | [min] | Run |
| 0.100 | [min] |  |
|  |  | PumpModule.Pump.Flow.Nominal: 0.900 [ml/min] |
|  |  | PumpModule.Pump.%B.Value: 10.0 [%] |
|  |  | PumpModule.Pump.Curve: 5 |

Thermo Scientific SII for Xcalibur Method

1.000 [min]

2.000 [min]

3.500 [min]

4.000 [min]

5.000 [min]

PumpModule.Pump.Flow.Nominal: 0.900 [ml/min]

PumpModule.Pump.%B.Value: 10.0 [%]

PumpModule.Pump.Curve: 5

PumpModule.Pump.Flow.Nominal: 0.900 [ml/min]

PumpModule.Pump.%B.Value: 98.0 [%]

PumpModule.Pump.Curve: 5

PumpModule.Pump.Flow.Nominal: 0.900 [ml/min]

PumpModule.Pump.%B.Value: 98.0 [%]

PumpModule.Pump.Curve: 5

PumpModule.Pump.Flow.Nominal: 0.900 [ml/min]

PumpModule.Pump.%B.Value: 10.0 [%]

PumpModule.Pump.Curve: 5

PumpModule.Pump.Flow.Nominal: 0.900 [ml/min]

PumpModule.Pump.%B.Value: 10.0 [%]

PumpModule.Pump.Curve: 5

5.000 [min] Stop Run

ColumnComp.CC_Temp.AcqOff PumpModule.Pump.Pump_Pressure.AcqOff

| **Method** Summary  Method Settings  Method Dur.atio,n (min): **5**  Global Parameters  Ion Source  Io,nSource Type: **H-ESI** Spr<ly Voltage: **Sta1iic** PositiveIon (V): **3501** NegativeIon (V): **2**5!®1)1  Current LCFlow (µUmin): **309**  Sheath Gas (Arb): **60**  Aux Gas (Arb): **15**  Sweep G.as (Arb): **2**  lo,n Tran&ferTubeTemp (0(): **35!0**  Vaporizer Temp (0(): **350**  APPi Lampe Nlo1itin **Use**  FAIMS Mode: Nlo1tl1ris1talledl  **MS Global SettingcS**  DefauIt Charge State: **1** Internal **Mass** Cc1libr.atiom **Off** Current Lock M.a&S: **Clurren1t**  Experiment#1 **[SRM]**  Start Time (min): **0**  End Time (min): **5**  **Master Scan:**  **SRM**  Polarity: **Posiliive**  Chromatographic Peak Width (sec): **6**  U!se Chromatogr<lphic Filter: **Tr,u�** | |
| --- | --- |
| U!seCycleTime: **TfiU** | **:e** |

Cycle Time (sec): **0.:6**

Points Per Peak: **10** U!seDwell Time F<lctor: **Fahe** U!seC-iiIibr<lted RF Lens: **True** 01Resolution (FWHM): **CH**

Q.3 Resolution (FWHM): **1.2**

CIDGas (mTorr): **1.5**

SourceFr<lgmentationi **25** U!seRetention Time Reference: **Fahe** Dirsplay Retention 1ime: **Tr,11:e** UseQuan lom **Fahe**

Show VirSuaIizatiom **Fahe**

**SRM Table**

| SRM Table | | | | | | |
| --- | --- | --- | --- | --- | --- | --- |
| **Compo1.111d** | **Retention Time(min)** | **RT Wi11dow (min)** | **Pirernrsor (m/z)** | **Pirod1.1ct (m/ z)** | **Collision E11ergy (V)** | **Min Dwell**  **Time (ms)** |
| Ondansetron | *3* | 4 | 294 | 170 | 27 | 198,025, |
| Ondansetron | *3* | 4 | 294 | 184 | 26 | 198,025, |
| Zolpidem | .3 | 4 | .308,1 | 23.5, | 35, | 198,025, |
